# Supplementary material for: Implications of successive blood feeding on Wolbachia-mediated dengue virus inhibition in Aedes aegypti mosquitoes
Source: Nat Commun. 2025 Jul 29;16:6971. doi: 10.1038/s41467-025-62352-2 (PMC12307751; doi:10.1038/s41467-025-62352-2)
Supplement: Supplementary file 1 — Supplementary Information [file 41467_2025_62352_MOESM1_ESM.pdf]

Implications of successive blood feeding on *Wolbachia*-mediated dengue virus inhibition in *Aedes aegypti* mosquitoes

Supplementary Information

Figures

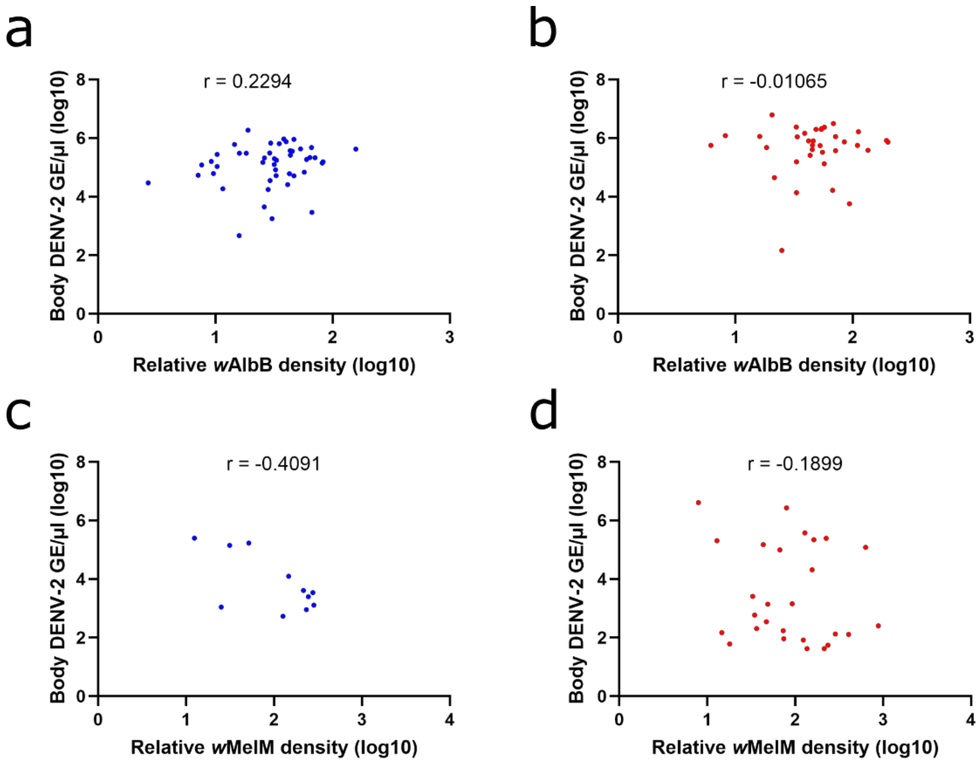

Figure S1: DENV-2 levels vs relative *Wolbachia* densities in bodies from individual single- and double-fed *w*AlbB and *w*MelM mosquitoes. a) No correlation for *w*AlbB single-fed mosquito bodies at 7 dpi. SF n = 46. **b)** No correlation for *w*AlbB double-fed mosquito bodies at 7 dpi. DF n = 35. **c)** No correlation for *w*MelM single-fed mosquito bodies at 7 dpi. SF n = 11. **d)** No correlation for *w*MelM double-fed mosquito bodies at 7 dpi. DF n = 27. Blue = single-fed, red = double-fed. The correlation between DENV-2 concentration and *Wolbachia* density was compared using two-tailed non-parametric Spearman correlation for each graph. r = correlation coefficient. Data was collected across 4 replicates for *w*AlbB groups and 5 replicates for *w*MelM groups. Source data are provided as a Source Data file.

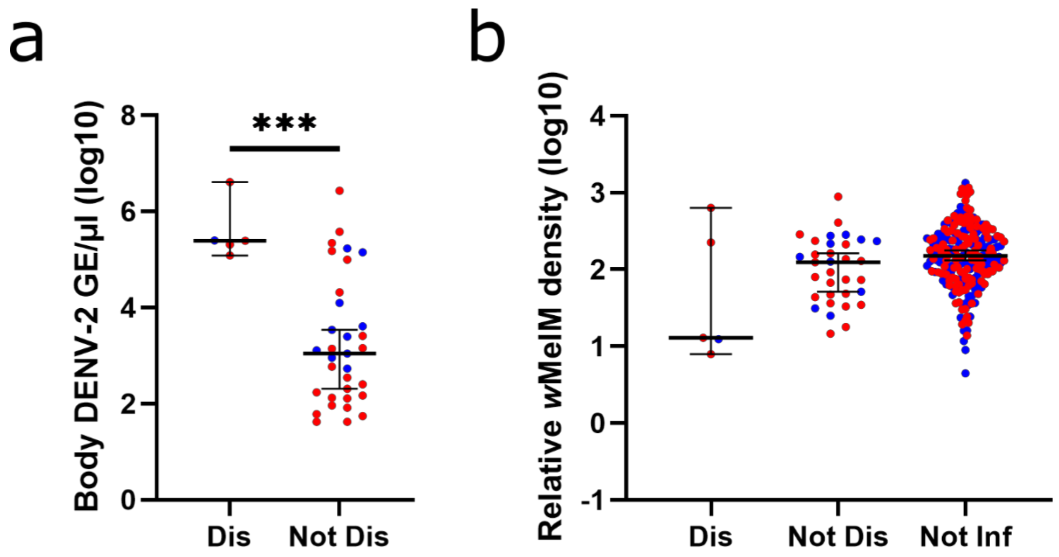

Figure S2: DENV-2 titers and relative *Wolbachia* density in *w*MelM mosquito bodies with disseminated and non-disseminated infection 7 dpi. a) Body DENV-2 titers in *w*MelM mosquitoes by feeding status. Total Dis n = 5 and Not Dis n = 33. Dis SF n = 1, Dis DF n = 4, Not Dis SF n = 10, and Not Dis DF n = 23. Dis vs Not Dis p = 0.0010. **b)** Relative body Wolbachia densities in *w*MelM mosquitoes by infection, dissemination, and feeding status. Total Dis n = 5, Not Dis n = 33, and Not Inf n = 229. Dis SF n = 1, Dis DF n = 4, Not Dis SF n = 10, Not Dis DF n = 23, Not Inf SF n = 108, and Not Inf DF n = 121. Comparisons were made using a two-tailed Mann-Whitney U test (**a**) or a Kruskal-Wallis test with Dunn's multiple comparisons (**b**). * = p ≤ 0.05, ** = p ≤ 0.01, *** = p ≤ 0.001, **** = p < 0.0001. Blue = single-fed, red = double-fed. Lines indicate median with 95% confidence interval. Data was collected across 5 replicates. Source data are provided as a Source Data file.

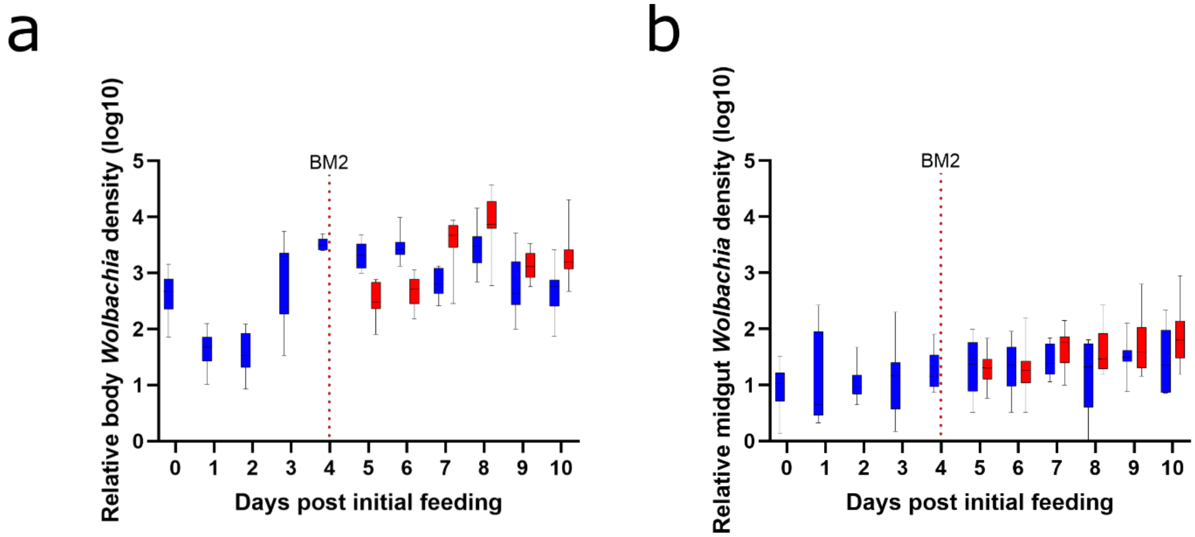

Figure S3: Time course of *Wolbachia* densities in single- and double-fed *w*AlbB mosquitoes. a) *w*AlbB Wolbachia densities in single- and double-fed whole mosquitoes following blood feeding. For each SF timepoint and for DF timepoints 5, 6, 7, and 9, n = 10. For DF day 8,

n = 9 and for DF day 10, n = 8. **b)** *w*AlbB Wolbachia densities in single- and double-fed mosquito midguts following blood feeding. For SF timepoints 0, 6, and 8 and for all DF timepoints except 10, n = 10. For SF timepoints 3, 4, 5, and DF day 10, n = 9. For SF days 2 and 10, n = 8. For SF days 1 and 9, n = 7 and for SF day 7, n = 6. Blue = single-fed, red = double-fed. Dashed red lines mark the timing of the second blood meal. Boxes denote first quartile and third quartile with a line at the median. Whiskers indicate minimum and maximum data points. Data for *Wolbachia* density time course graphs were collected over 1 replicate. Source data are provided as a Source Data file.

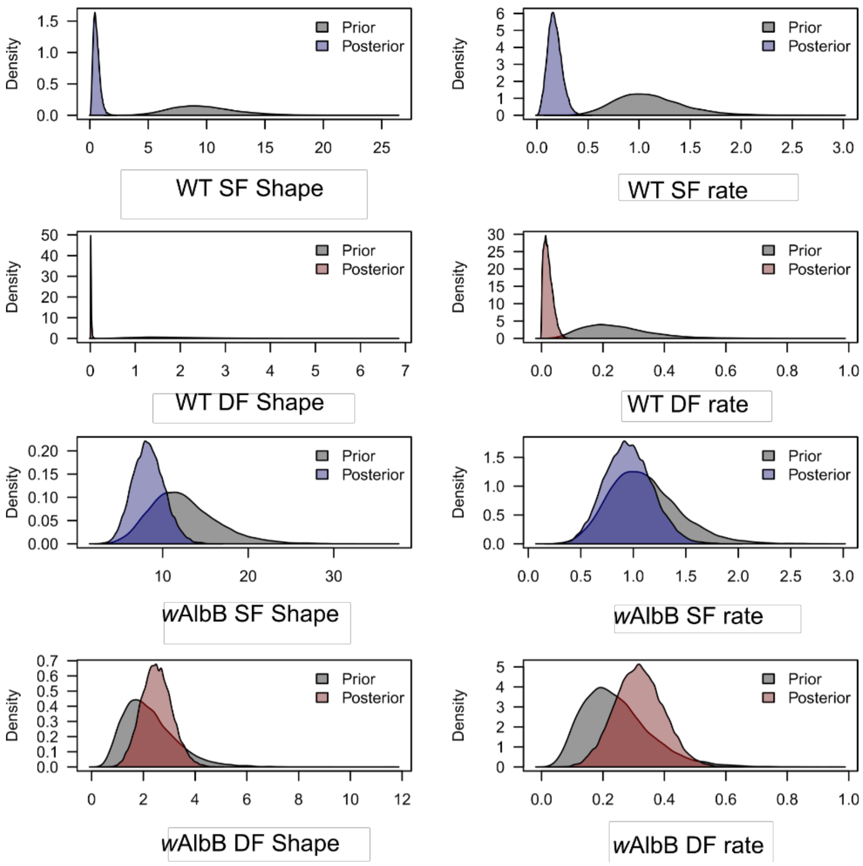

Figure S4: Prior and posterior estimates of shape and rate for gamma-distribution model of dpi for DENV-2 dissemination to the mosquito salivary glands. Blue = single-fed, red = double-fed, gray = prior. WT = wildtype mosquitoes without *Wolbachia*

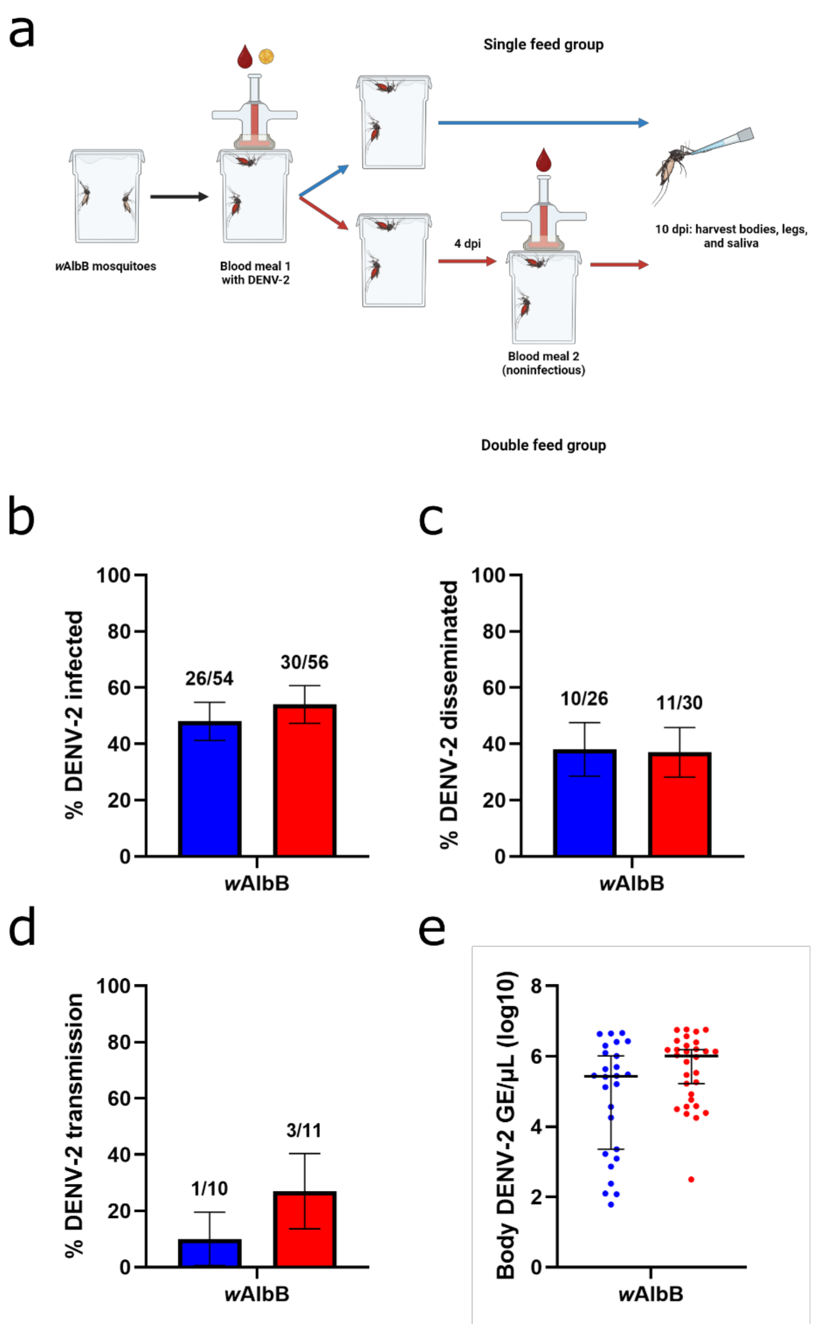

Figure S5: Rates of infection, dissemination, and saliva transmission of DENV-2 in *w*AlbB mosquitoes at 10 dpi. a) Experimental design for saliva transmission studies of single- and double-fed *w*AlbB mosquitoes. *Created in BioRender. Brackney, D. (2025) https://BioRender.com/hj4as8r* **b)** Proportion of infected single- and double-fed *w*AlbB mosquitoes at 10 dpi. Numbers indicate infected mosquitoes over total fed mosquitoes. **c)** Proportion of single- and double-fed *w*AlbB mosquitoes with disseminated DENV-2 infection at 10 dpi. Numbers indicate mosquitoes with disseminated infection (measured using legs) over infected mosquitoes. **d)** Proportion of single- and double-fed *w*AlbB mosquitoes with DENV-2 in the saliva at 10 dpi. Numbers indicate mosquitoes with DENV-2 in collected saliva (indicating transmission ability)

over mosquitoes with a disseminated infection. **e)** DENV-2 titers in single- and double-fed *w*AlbB mosquito bodies at 10 dpi. Total SF n = 26 and DF n = 30. Comparisons were made using two-sided Fisher's exact tests (**b-d**) or a two-tailed Mann-Whitney U test (**e**). * = p ≤ 0.05, ** = p ≤ 0.01, *** = p ≤ 0.001, **** = p < 0.0001. Blue = single-fed, red = double-fed. For **b-d**, lines indicate mean ± standard error of the mean of the total sample proportions. For **e**, lines indicate median with 95% confidence interval. Salivation data was collected from 1 replicate in *w*AlbB mosquitoes. Source data for **b-e** are provided as a Source Data file.

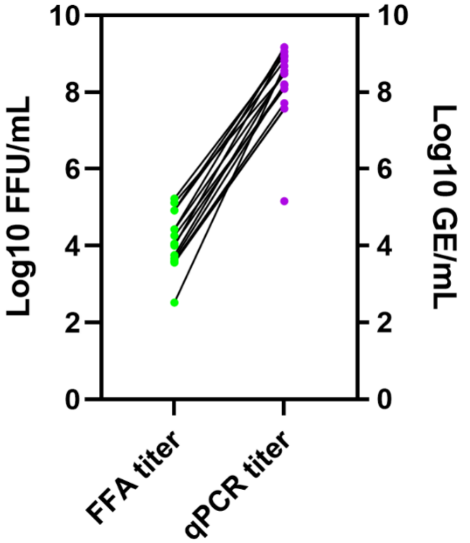

Figure S6: Comparison of DENV-2 concentrations via FFA or RT-qPCR. Log10 DENV-2 body concentrations in selected samples at 7 dpi as measured by FFA vs measurements through RT-qPCR. FFA n = 15 and qPCR n = 17. A subset of samples were analyzed across 4 replicates and both WT and *w*AlbB groups. Source data are provided as a Source Data file.

Tables

Model	Marginal likelihood	Bayes factors
1: wAlbB/WT x SF/DF	-70.93477	BF1>2 = 2.3x104
2: wAlbB/WT only	-80.97609	BF2>3 = 1.4x1047
3: SF/DF only	-189.5368	BF3>4 = 11.3
4: No distinction among mosquitoes	-191.9592	

Table S1: Marginal likelihood and Bayes factors for models of dpi for DENV-2 dissemination to the mosquito salivary glands.

Experiment	Dilution	GE/mL	FFU/mL	Figure showing data
Infection and dissemination rep 1	1:5	2.03E+06	3.00E+05	1, 2, S1, S2, S6
Infection and dissemination rep 2	1:5	1.67E+06	2.00E+05	1, 2, S1, S2, S6
Infection and dissemination rep 3	1:5	2.26E+07	1.70E+07	1, 2, S1, S2, S6
Infection and dissemination rep 4	1:5	6.30E+06	1.33E+06	1, 2, S1, S2, S6
Infection and dissemination rep 5 wMelM	1:5	2.28E+07	5.00E+06	1, 2, S1, S2, S6
Salivation experiments wAlbB	1:5	2.88E+07	9.33E+06	S5
Time course rep 1	1:12	3.35E+06	2.33E+05	3, 4
Time course rep 2	1:12	7.08E+07	4.00E+07	3, 4

Table S2: DENV-2 titers given to mosquitoes as measured by qPCR (GE/mL) and FFA (FFU/mL).

**Name**	**Sequence 5’->3’**	**Use**	**Source**
DENV-2_F	CATGGCCCTKGTGGCG	DENV-2 titer RT-qPCR	1
DENV-2_R	CCCCATCTYTTCAGTATCCCTG	DENV-2 titer RT-qPCR	1
DENV-2_P	FAM-TCCTTCGTTTCCTAACAATCC-BHQ-1	DENV-2 titer RT-qPCR	1
wMwA-F	GAAGTTGAAGCACAGTGTACCTT	Wolbachia abundance qPCR	2
wMwA-R	GCTTGATATTCCTGTAGATTCATC	Wolbachia abundance qPCR	2
AeAegypti-aRpS6-F	ATCAAGAAGCGCCGTGTCG	Ae. aegypti housekeeping gene qPCR	3
AeAegypti-aRpS6-R	CAGGTGCAGGATCTTCATGTATTCG	Ae. aegypti housekeeping gene qPCR	3

Table S3: Primers used to measure DENV-2 titers, *Wolbachia* abundance, and *Ae. aegypti* housekeeping gene copies.

Supplemental References

1. Callahan, J. D. *et al.* Development and evaluation of serotype- and group-specific fluorogenic reverse transcriptase PCR (TaqMan) assays for dengue virus. *J. Clin. Microbiol.* **39**, 4119–4124 (2001).
2. Lau, M. J., Hoffmann, A. A. & Endersby-Harshman, N. M. A diagnostic primer pair to distinguish between wMel and wAlbB Wolbachia infections. *PLoS One* **16**, 1–11 (2021).
3. Lee, S. F., White, V. L., Weeks, A. R., Hoffmann, A. A. & Endersby, N. M. High-throughput PCR assays to monitor Wolbachia infection in the dengue mosquito (Aedes aegypti) and Drosophila simulans. *Appl. Environ. Microbiol.* **78**, 4740–4743 (2012).
