# Supplementary material for: Implications of successive blood feeding on Wolbachia-mediated dengue virus inhibition in Aedes aegypti mosquitoes
Source: Nat Commun. 2025 Jul 29;16:6971. doi: 10.1038/s41467-025-62352-2 (PMC12307751; doi:10.1038/s41467-025-62352-2)
Supplement: Supplementary file 3 — Reporting Summary [file 41467_2025_62352_MOESM3_ESM.pdf]

nature portfolio

Corresponding author(s): Rebecca M. Johnson, Doug E. Brackney, Chantal B.F. Vogels

Last updated by author(s): Jul 1, 2025

Reporting Summary

Nature Portfolio wishes to improve the reproducibility of the work that we publish. This form provides structure for consistency and transparency in reporting. For further information on Nature Portfolio policies, see our Editorial Policies and the Editorial Policy Checklist.

Statistics

For all statistical analyses, confirm that the following items are present in the figure legend, table legend, main text, or Methods section.

n/a	Confirmed	
☐	☒	The exact sample size (*n*) for each experimental group/condition, given as a discrete number and unit of measurement
☐	☒	A statement on whether measurements were taken from distinct samples or whether the same sample was measured repeatedly
☐	☒	The statistical test(s) used AND whether they are one- or two-sided<br>*Only common tests should be described solely by name; describe more complex techniques in the Methods section.*
☐	☒	A description of all covariates tested
☐	☒	A description of any assumptions or corrections, such as tests of normality and adjustment for multiple comparisons
☐	☒	A full description of the statistical parameters including central tendency (e.g. means) or other basic estimates (e.g. regression coefficient) AND variation (e.g. standard deviation) or associated estimates of uncertainty (e.g. confidence intervals)
☐	☒	For null hypothesis testing, the test statistic (e.g. F, t, r) with confidence intervals, effect sizes, degrees of freedom and P value noted<br>*Give P values as exact values whenever suitable.*
☐	☒	For Bayesian analysis, information on the choice of priors and Markov chain Monte Carlo settings
☐	☒	For hierarchical and complex designs, identification of the appropriate level for tests and full reporting of outcomes
☐	☒	Estimates of effect sizes (e.g. Cohen's *d*, Pearson's *r*), indicating how they were calculated

Our web collection on statistics for biologists contains articles on many of the points above.

Software and code

Policy information about availability of computer code

Data collection	Bio-Rad CFX Maestro Software was used to collect RT-qPCR data
Data analysis	Custom code used for data analysis is available on GitHub at: https://github.com/TAlexPerkins/doubleFeedWolbachia

For manuscripts utilizing custom algorithms or software that are central to the research but not yet described in published literature, software must be made available to editors and reviewers. We strongly encourage code deposition in a community repository (e.g. GitHub). See the Nature Portfolio guidelines for submitting code & software for further information.

Data

Policy information about availability of data

All manuscripts must include a data availability statement. This statement should provide the following information, where applicable:

- Accession codes, unique identifiers, or web links for publicly available datasets
- A description of any restrictions on data availability
- For clinical datasets or third party data, please ensure that the statement adheres to our policy

All data are included in this manuscript and the supplementary files. Source data are provided with this paper.

Research involving human participants, their data, or biological material

Policy information about studies with human participants or human data. See also policy information about sex, gender (identity/presentation), and sexual orientation and race, ethnicity and racism.

Reporting on sex and gender	N/A
Reporting on race, ethnicity, or other socially relevant groupings	N/A
Population characteristics	N/A
Recruitment	N/A
Ethics oversight	N/A

Note that full information on the approval of the study protocol must also be provided in the manuscript.

Field-specific reporting

Please select the one below that is the best fit for your research. If you are not sure, read the appropriate sections before making your selection.

☐ Life sciences ☐ Behavioural & social sciences ☒ Ecological, evolutionary & environmental sciences

For a reference copy of the document with all sections, see nature.com/documents/nr-reporting-summary-flat.pdf

Ecological, evolutionary & environmental sciences study design

All studies must disclose on these points even when the disclosure is negative.

Study description	We determined the impact of successive blood feeding on dengue virus infection and dissemination in Aedes aegypti mosquitoes in presence and absence of Wolbachia (wAlbB and wMelM strains).
Research sample	Groups of WT, wAlbB, and wMelM Aedes aegypti mosquitoes were fed a dengue virus (serotype 2) infectious blood meal and split in "single-fed" and "double-fed" groups. The double-fed group received a second non-infectious blood meal at day 4. Dengue virus infection and dissemination was determined after 5-10 days incubation via RT-qPCR.
Sampling strategy	We used similar sample sizes for vector competence experiments following the established precedence in literature.
Data collection	Infection and dissemination was determined from mosquito bodies and legs, respectively, using RT-qPCR. A subset of RT-qPCR results were compared to results from focus forming assays.
Timing and spatial scale	Data were collected over several independent replicates as specified in the manuscript for each experiment.
Data exclusions	No data were excluded from the analysis
Reproducibility	Experiments were replicated 1-5 times to reach sufficient sample sizes.
Randomization	For each replicate, all mosquitoes were from the same cohort. Engorged mosquitoes were randomly split in "single-fed" and "double-fed" groups.
Blinding	N/A

Did the study involve field work? ☐ Yes ☒ No

Reporting for specific materials, systems and methods

We require information from authors about some types of materials, experimental systems and methods used in many studies. Here, indicate whether each material, system or method listed is relevant to your study. If you are not sure if a list item applies to your research, read the appropriate section before selecting a response.

Materials & experimental systems

n/a	Involved in the study
☐	☒ Antibodies
☐	☒ Eukaryotic cell lines
☒	☐ Palaeontology and archaeology
☐	☒ Animals and other organisms
☒	☐ Clinical data
☒	☐ Dual use research of concern
☒	☐ Plants

Methods

n/a	Involved in the study
☒	☐ ChIP-seq
☒	☐ Flow cytometry
☒	☐ MRI-based neuroimaging

Antibodies

Antibodies used	Mouse anti-flavivirus group antigen antibody from NovusBio D1-4G2-4-15 (4G2) was used at a dilution of 1:500 and Invitrogen goat anti-mouse IgG (H+L) cross-adsorbed secondary antibody, Alexa Fluor 488 was used a dilution of 1:200.
Validation	Validation was done by manufacturers.

Eukaryotic cell lines

Policy information about cell lines and Sex and Gender in Research

Cell line source(s)	All mosquito and vertebrate cell lines used in these studies were acquired from ATCC.
Authentication	Cell lines were authenticated by ATCC.
Mycoplasma contamination	The cell lines are routinely monitored for mycoplasma contamination and the cell lines used in this study tested negative for mycoplasma.
Commonly misidentified lines (See ICLAC register)	None of the cell lines used are commonly misidentified.

Animals and other research organisms

Policy information about studies involving animals; ARRIVE guidelines recommended for reporting animal research, and Sex and Gender in Research

Laboratory animals	Aedes aegypti (collected from Cairns, Queensland, Australia) wildtype (not transinfected), wAlbB (transinfected with wAlbB Wolbachia strain), and wMelM (transinfected with wMelM Wolbachia strain).
Wild animals	N/A
Reporting on sex	N/A
Field-collected samples	N/A
Ethics oversight	Ethical approval is not required for experiments involving mosquitoes.

Note that full information on the approval of the study protocol must also be provided in the manuscript.

Plants

Seed stocks	N/A
Novel plant genotypes	N/A
Authentication	N/A
